# Case Report: Invasive fungal infection after anti-CD19 CAR-T cell therapy. Implication for antifungal prophylaxis

**DOI:** 10.3389/fimmu.2023.1272798

**Published:** 2023-09-28

**Authors:** Elsa Pennese, Prassede Salutari, Luigi Carriero, Francesco Restuccia, Antonio Fabio De Filippis, Giulia De Luca, Raffaella Giancola, Francesco Guardalupi, Giulia Corradi, Bianca Fabi, Stefano Baldoni, Mauro Di Ianni

**Affiliations:** ^1^ Hematology Unit, Department of Oncology and Hematology, Spirito Santo Hospital, Pescara, Italy; ^2^ Thoracic Surgery Unit, Spirito Santo Hospital, Pescara, Italy; ^3^ Department of Medicine and Aging Sciences, University of Chieti-Pescara, Chieti, Italy

**Keywords:** CAR-T cell therapy, invasive aspergillosis, case report, immunotherapy, antifungal prophylaxis

## Abstract

CAR-T therapy has revolutionized the treatment of relapsed/refractory B-cell malignancies. Patients who are receiving such therapy are susceptible to an increased incidence of infections due to post-treatment immunosuppression. The need for antifungal prophylaxis during the period of neutropenia remains to be determined. The clinical outcome of a 55-year-old patient with relapsed/refractory DLBCL who received axicabtagene ciloleucel is described here. The patient developed CRS grade II and ICANS grade IV requiring tocilizumab, prolonged use of steroids and anakinra. An invasive pulmonary aspergillosis arose after 1 month from CAR-T reinfusion, resolved with tracheal sleeve pneumonectomy. The patient is now in Complete Remission. This case suggests that antifungal prophylaxis should be considered. We have now included micafungin as a standard prophylaxis in our institution.

## Introduction

Therapy with chimeric antigen receptor T cells directed against the CD19 antigen (CD19 CAR-T) has substantially changed the outcomes of the patients with relapsed/refractory B-cell lymphoma (1–3). CAR T-related side effects include cytokine release syndrome (CRS) and immune effector cell-associated neurotoxicity syndrome (ICANS), which, together with persistent neutropenia, B-cell aplasia and subsequent hypogammaglobulinemia create a period of profound immunosuppression which predisposes these patients to a high risk of infectious complications (4, 5). There is currently no strong recommendation for the use of antifungal prophylaxis in this patient setting (6). Here, we report on a 55-year-old woman with chemorefractory DLBCL who developed an invasive pulmonary aspergillosis treated and resolved surgically (7). Based on the present case we are now including antifungal prophylaxis as a standard treatment in the patient who underwent CAR-T therapy.

## Case presentation

**Figure 1:**
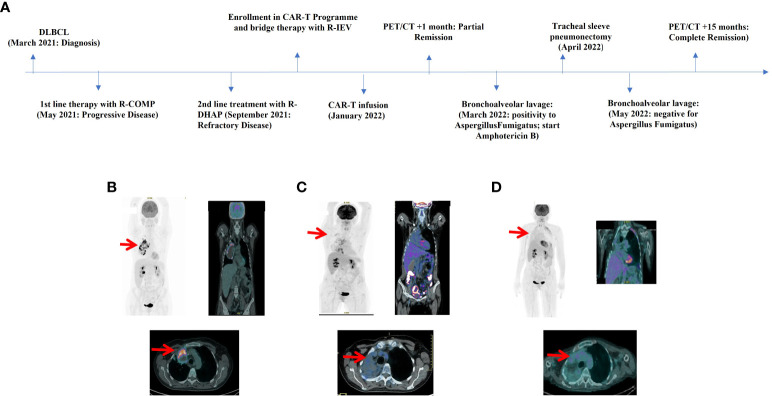
**(A)** Timeline illustrating the therapies carried out and the outcomes. R-COMP: Prednisone, Cyclophosphamide, Vincristine, Liposomal Doxorubicin and Rituximab; R-DHAP, Rituximab, Dexamethasone, Cytarabine and Cisplatin; R-IEV, Rituximab, Ifosfamide, Epirubicin and Etoposide. **(B)** FDG-PET/CT image pre-CAR-T infusion revealed a hypermetabolic activity in the right lung and mediastinum (SUV max 18.4) with necrosis of the mediastinal mass. **(C)** FDG**-**PET/CT image at +30 days after CAR-T infusion showed partial response (PR) to therapy according to Lugano and Lyric criteria (SUVmax 4.9) with a new area of hypermetabolic activity in right lung and bronchus of infectious origin. **(D)** FDG-PET/CT image at +15 months demonstrated complete response (CR) to therapy according to Lugano and Lyric criteria. No hypermetabolic activity of infectious origin.

**Figure 2:**
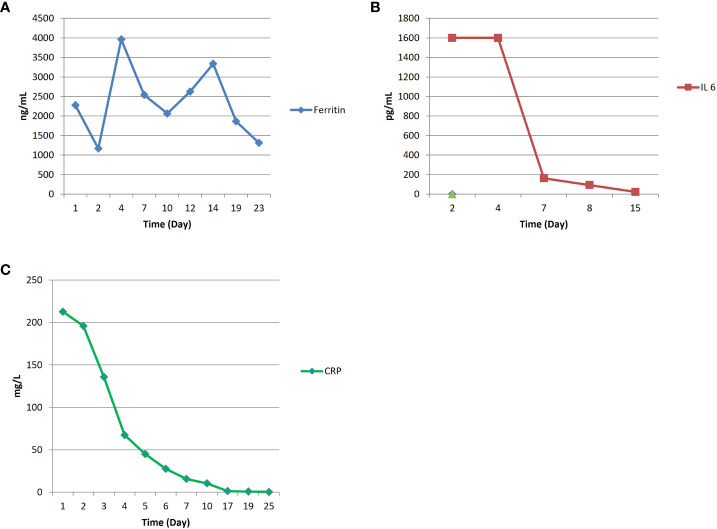
Monitoring of diagnostic CRS parameters including ferritin **(A)**, IL-6 **(B)** and CRP **(C)**.

**Figure 3:**
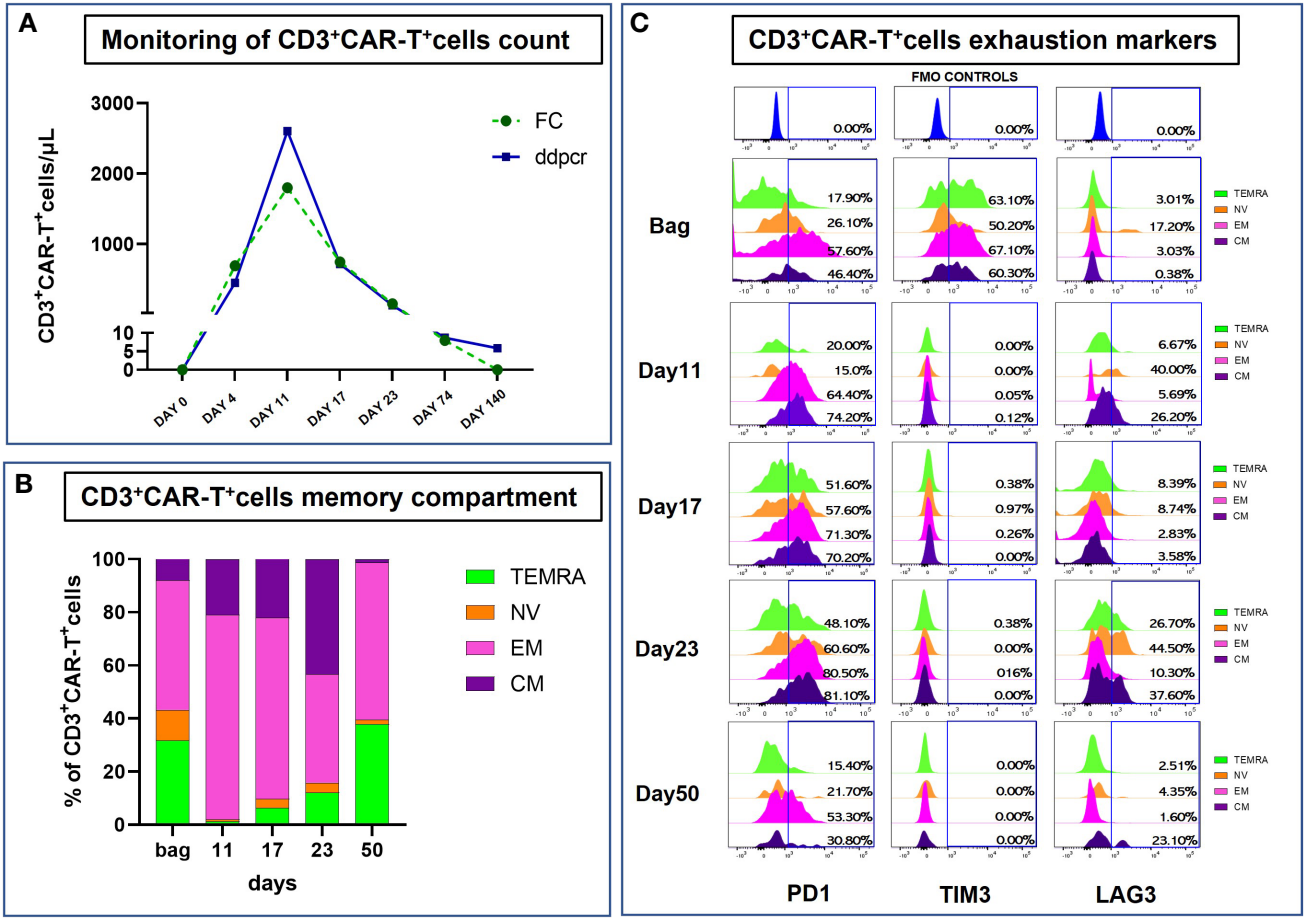
Monitoring of infused CART+CD3+ cells. **(A)** Analysis of CAR T+CD3+ cells from patient-derived peripheral blood (PB) samples collected at different timepoints after infusion using both ddPCR and Flow Cytometry (expressed as CAR-T cells/μL). **(A)** For Flow Cytometry absolute count, PB-samples were stained with the following antibodies, as recommended by manufacturer’s instructions: anti-CD45 (clone: 2D1), anti-CD3 (clone: SK7), anti-CD4 (clone: RPA-T4), anti-CD8 (clone: RPA-T8), anti-CD19 (clone: SJ25C1) and anti-CD16/CD56 (clone: 3G8/NCAM16.2) from BD Biosciences (BD Biosciences, La Jolla, CA); anti-CD19CAR (130-129-550) from Miltenyi Biotec (Miltenyi Biotech, Bergisch Gladbach Germany). Fluorescence Minus One (FMO) controls were used to set the background signals and to identify and gate the positive CD19CAR+ cells population on total CD3+T cells. Data acquisition was performed using BD FACS Lyric™ instrument (BD Biosciences) and data were analyzed with the BD FACSuite™ software version 1.4 with predefined and optimized settings for CAR T-cell analysis. A total of 300 ng of DNA extracted from peripheral blood was used for digital droplet PCR (ddPCR) analysis for CAR-T+ cell detection. The CAR-T CD-19 test was used according to the specific protocol (dEXD88164642 Bio-Rad, Hercules, CA). **(B, C)** Flow cytometry analysis of CART+CD3+ cells Memory Compartment and Exhaustion markers from post-infusion bag to day+50 collected samples. Briefly, cells were labeled with the following set of antibodies: anti-CD19CAR (130-129-550), anti-PD-1 (clone: REA802) and anti-TIM-3 (clone: F38-2E2) from Miltenyi Biotec; anti-CD45 (clone: 2D1), anti-CD3 (clone: SK7), anti-CD4 (clone: RPA-T4), anti-CD8 (clone: RPA-T8), anti-CD197 (clone: 150503), 7-AAD, anti-CD45RA (clone: HI100), anti-LAG-3 (clone: T47-530), anti-CD25 (clone: 2A3) and anti-HLA-DR (clone: G46-6) from BD Biosciences. Data acquisition was performed using BD FACS ARIA III™ instrument (BD Biosciences) and data were analyzed with the BD FACSDiva™ software version 9.0.1.

A 55 years old woman diagnosed with diffuse large B cell lymphoma (DLBCL) with multiple involvement of the right lung, had persistent disease after three prior lines of treatments, including anti-CD20 antibody, alkylating agents, anthracycline and antimetabolites. She had no other relevant past medical history, with an ECOG (Eastern Cooperative Oncology Group) Performance Status score of 1 and no organ dysfunctions including normal left-ventricular ejection fraction. She was not candidate for an autologous stem cell transplant because of refractory disease and she was referred for treatment with CD19 CAR-T cell therapy using axicabtagene ciloleucel (**Figure 1A**). PET/CT scan revealed a hypermetabolic activity in the right lung and within the mediastinum (**Figure 1B**). The patient received conditioning chemotherapy consisting of fludarabine and cyclophosphamide followed by the infusion of 144x10^6^ CD3+ T cells (80.8% CD19-CAR-T positive; 3,8:1 CD4:CD8 ratio). CAR-HAMATOTOX (HT) score was 3 (high risk) (7). Anti-viral (acyclovir 400 mg bid) and antibacterial prophylaxis (levofloxacin 750 mg/day) was performed during neutropenia (ANC < 0.5 x 10^9^/L > 7 days), until neutrophil recovery (day +10). On day +1, the patient developed fever with unstable cardiopulmonary function and concomitant IL-6 arising (**Figure 2**), which was classified as CRS grading 3 according to ASCT consensus grading (8). Patient received tocilizumab (2 doses) followed by corticosteroids (dexamethasone 10 mg every 6 hours). Starting from day +6 the patient worsened neurologically falling into a coma and allowing us to diagnose ICANS grade IV. The patient was referred to the Intensive Care Unit (ICU), and high dose of corticosteroids (methylprednisolone 100 mg daily for 3 days) and Anakinra (1000 mg daily for 7 days) were started. Brain Nuclear Magnetic Resonance showed no pathological changes. Analysis of cerebrospinal fluid (CSF) revealed the presence of T cells (4 cells/µl) 97.4% of which expressed the CD19 CAR construct. The presence of CAR-T in the cerebrospinal fluid was examined weekly for one month, until it was negative on day + 30. At each lumbar puncture, steroids were administered. No signs of infections or lymphoma meningeal involvement was detected. Patient’s neurological status improved on day 15, and she began a steroid taper. ICANS ended on day +24. PET/CT performed on day +30 showed partial response (PR) to CAR-T treatment (9) (**Figure 1C**). Despite that, the patients displays fever, persistent cough with an episode of haemoftoe. A bronchoscopy with bronchoalveolar lavage was positive for Asperigillus Fumigatus; the chest CT scan showed an invasive fungal pneumonia, with total collapse of the right middle and upper lobes of the lung. Antifungal treatment with liposomal amphotericin B was began on day +35. A subsequent bronchoscopic showed several endotracheal fungal vegetations and widespread necrosis of the right bronchus extending to the middle and superior lobes. No pathological findings in the left system. Bronchoscopic biopsies showed no malignancy but only fungal hyphae. We started amphotericin B empirically before the arrival of the result of the bronchoalveolar lavage and we continued it as it had worked by preventing infection in the contralateral lung. Droplet digital polymerase chain reaction (dd-PCR) analysis showed the presence of CAR-T DNA in the bronchoscopic material (allelic ratio 0.17%). CAR-T cells monitoring performed by flow cytometry analysis and ddPCR on peripheral blood showed a peak on day +11 from infusion (1800CAR-T cell/µl; 2605CAR-T cell/µl of gDNA at ddPCR). A right tracheal sleeve pneumonectomy was performed on day +60 in another institution (10). Monitoring showed negativization of CD3/CAR-T on day +170 by flow cytometry, when there was still positivity in ddPCR (5.9 cell/µl of gDNA**)** (**Figure 3A**). Analysis of naïve (N), Central Memory (CM), Effector Memory (EM) and Effector Memory re-expressing CD45RA (TEMRA) CD3^+^CAR-T^+^ subsets showed a prevalent population of EM T cells (**Figure 3B**). Exhaustion markers (PD1, TIM3 and LAG3) examined in all CAR-T cell subsets up to day +50 showed a constant expression over the time of the PD1 with a reduction of the expression of TIM3 and LAG3 in comparison with infused cells (**Figure 3C**). The patient is now in Complete Remission (CR) at last PET/TC scan (+ 15 months) (**Figure 1D**). The patient is now enjoying her life and reports well-being.

## Discussion and conclusions

Post CAR-T infectious complications occur with a frequency of 23% in the first month and 14% up to 3 months. Bacterial infections are the most frequent in the first month, while viral infections are the predominant ones after the first 30 days (8). Invasive fungal infections, on the other hand, are rare (1-7%) frequently life-threatening and are associated with severe neutropenia and CRS (8, 11). The risk factors for fungal infections are not completely defined in this cohort and therefore there is no consensus about the favored choice and duration of antifungal prophylaxis after CAR-T-cell therapy. Prolonged use of steroids remains the major infectious risk factor (12) due to the fact that steroids negatively regulate innate and adaptative immunity and also modulate the inflammatory response to different pathogens (13). No other additional risk factors for infections beyond prolonged neutropenia (ANC < 0.5 X 10^9^/L > 7 days) severe hypogammaglobulinaemia and disease localization in the right lung are present in our case. With several recommendations in place for CAR T-cell treatments, the appropriate management of infectious consequences remains a clinical challenge (14–20). It is not currently known whether certain subgroups of CAR-T-cell recipients may benefit from anti-mould prophylaxis. Guidelines suggest fluconazole or micafungin prophylaxis against Candida spp during neutropenia is a good approach (18). The CAR-HEMATOTOX score represents an effective and clinically feasible score to risk-stratify patients for hematological toxicity, infectious complications, and clinical outcomes prior to lymphodepletion (7). Large multicenter prospective studies are required to establish best practice for prevention and management Invasive Fungal Infections (IFIs) in this susceptible population. Based on the superior efficacy of micafungin to that of fluconazole (21) we, as a policy of the center, have now included antifungal primary prophylaxis with micafungin as a standard treatment in the patient who underwent CAR-T therapy.

## Data availability statement

The original contributions presented in the study are included in the article/supplementary material. Further inquiries can be directed to the corresponding author.

## Ethics statement

The studies involving humans were approved by Hematology Unit Internal Review Board. The studies were conducted in accordance with the local legislation and institutional requirements. The participants provided their written informed consent to participate in this study. Written informed consent was obtained from the individual(s) for the publication of any potentially identifiable images or data included in this article. Written informed consent was obtained from the participant/patient(s) for the publication of this case report.

## Author contributions

EP: Investigation, Writing – original draft, Writing – review & editing, Data curation, Formal Analysis, Funding acquisition. PS: Data curation, Investigation, Writing – review & editing, Supervision. LC: Data curation, Investigation, Methodology, Writing – review & editing. FR: Data curation, Investigation, Methodology, Writing – review & editing. AD: Data curation, Investigation, Methodology, Writing – review & editing. GD: Data curation, Investigation, Writing – review & editing. RG: Formal Analysis, Methodology, Writing – review & editing. FG: Formal Analysis, Data curation, Writing – review & editing. GC: Methodology, Validation, Writing – review & editing. BF: Methodology, Formal Analysis, Writing – review & editing. SB: Formal Analysis, Methodology, Data curation, Writing – review & editing. MD: Formal Analysis, Conceptualization, Funding acquisition, Investigation, Writing – original draft, Writing – review & editing.
